# A narrative review of undergraduate peer-based healthcare ethics teaching

**DOI:** 10.5116/ijme.5650.54ad

**Published:** 2015-12-12

**Authors:** Thomas Hindmarch, Silvia Allikmets, Felicity Knights

**Affiliations:** 1 Emergency Department, Epsom and St Helier's University Hospitals NHS Trust, UK; 2 School of Medicine, King's College London, UK; 3 General Paediatrics, Newcastle-upon-Tyne NHS Foundation Trust, UK

**Keywords:** Peer teaching, ethics teaching, medical student, professionalism teaching

## Abstract

**Objectives:**

This study explores the literature in establishing the value of undergraduate peer-based healthcare ethics teaching as an educational methodology.

**Methods:**

A narrative review of the literature concerning peer-based ethics teaching was conducted. MEDLINE, EMBASE, CINAHL, SCOPUS databases, and the Cochrane Library, were systematically searched for studies of peer-based ethics or professionalism teaching. Selected studies related peer-based teaching to ethics education outcomes.

**Results:**

Ten publications were identified. Selected studies were varied in their chosen intervention methodology and analysis. Collectively, the identified studies suggest peer-based ethics education is an effective and valued educational methodology in training healthcare professionals. One paper suggests peer-based ethics teaching has advantages over traditional didactic methods. Peer-based ethics teaching also receives positive feedback from student participants. However, the limited literature base demonstrates a clear need for more evaluation of this pedagogy.

**Conclusions:**

The current literature base suggests that undergraduate peer based healthcare ethics teaching is valuable in terms of efficacy and student satisfaction. We conclude that the medical community should invest in further study in order to capitalise upon the potential of peer-based ethics teaching in undergraduate healthcare education.

## Introduction

The role of ethics in medicine has been integral to practice for centuries; the ancient Hippocratic oath itself forms the basis of modern day core principles such as ‘do no harm’ and the respect of patient confidentiality. However, widely accepted defining ethical principles were only formally set out surprisingly recently in Beauchamp and Childress’ Principles of Biomedical Ethics.^1^ Furthermore, ethics as a core component of the medical curriculum is a relatively new concept.^2^ In the modern era, the breadth and depth of ethical knowledge required by clinicians is extensive; the World Medical Association International Code of Medical Ethics acts as an example of the widespread duties to which physicians are expected to adhere.^3^ Furthermore, ethical responsibilities are more formally expanding throughout healthcare teams with nursing staff and allied health professionals producing their own ethical guidance.^4^ Although the importance of ethical education is now widely recognised, methodologies of teaching remain varied.^5^

An intervention that has been successful as an educational methodology in other areas (including healthcare curricula) is peer-to-peer teaching.^6^^-^^8^ A 2007 review of peer-to-peer teaching cited various justifications for the use of such teaching methods, including: motivation for students, establishing a comfortable learning environment, the development of strong leadership and instruction skills, and to alleviate pressure on faculty members to increase their teaching.^9^ Yu et al. conducted a similar review on the application of peer-assisted learning in medical school environments, concluding that peer-to-peer teaching was as successful as traditional teacher-student methods. The review advocated for the integration of peer-based teaching into medical school curricula, as it would be an effective means to teach the growing population of medical students.^10^ Wong et al. focused on the impact that teaching had on the peer educator tracking their academic success compared to a control group of non-peer educators. Peer educator academic performance was enhanced; a finding that suggests such practice could be mutually beneficial.^11^

Ethics education has become a core component of medical education in recent times with methods of teaching ethics varying between educational establishments. Given the success of peer-based teaching in the broader medical education setting, one would anticipate similarly positive outcomes in ethics education. Furthermore, the use of peer teaching creates a seemingly ideal setting for discussion of ethics, a topic typified by sharing of ideas rather than binary solutions. Rahim et al. point out that learning outcomes from this teaching format extend beyond the curriculum with positive role modelling and sharing of individual scenarios enhancing the educative experience.^12^ Consequently, our review sought to establish the role of peer-based ethics teaching in the healthcare community and whether it constitutes a viable and valuable teaching methodology in ethics education.

## Methods

A search was prospectively designed to locate all the literature relevant to achieving the aims of reviewing the value of peer based ethics teaching. Ethical approval was not deemed necessary, as our study constitutes a narrative review and involved no primary data such as individual patient data collection or analysis. Pilot reviews were conducted to ascertain the volume of literature relating to our search question. During these pilots we found literature in which ethical education is situated under a wider teaching programme that is defined as professionalism. For example, Elliot et al. describe a stream of their curriculum entitled ‘professionalism and the practice of medicine’, that incorporates specific emphasis on ethical judgment and utilizes a peer assisted learning approach in achieving this.^13^ Therefore we decided to include the search terms profession, professional and professionalism, when exploring the literature and to split the literature generated based on the pedagogical approach taken: 1) Direct peer-to-peer ethics education and 2) Indirect peer-to-peer ethics education. Those in the direct category are studies in which ethical education outcomes are explicit in the study design. Conversely, the indirect category accounts for studies in which achieving ethical outcomes forms part of the wider professionalism education outcomes and is thus less explicit.

In capturing the relevant literature to our aims, we also found it necessary to define clearly our understanding of a peer-based teaching approach. Many medical educators cite Keith Topping’s definition of peer-assisted learning when exploring this teaching methodology. He defines peer-assisted learning as ‘people from similar social groupings who are not professional teachers helping each other to learn and learning themselves by teaching’.^14^ However, in our pilot reviews, it emerged that a varied range of study methodologies relevant to our study question exists. Consequently, as authors, we wanted to broaden the focus away from peer-assisted learning to any intervention in which peers play a significant role in the educational development of fellow students. Resultantly, in the context of this article, a peer-based teaching approach refers to any educational intervention in which students teach other students, offer peer-assessment or feedback, or learn or attempt to learn with other peers in a collaborative manner.^15^ This broader definition accommodates the heterogeneous methodologies utilised within the literature, facilitating the capture of all literature relevant to our study question.

When weighing the literature generated to form narrative conclusions, we gave greater emphasis to studies using statistical analysis or formal measurement of outcome, and therefore, analysis has been reported study by study according to perceived strength of outcome.

Inclusion criteria required that selected papers: 1) were in the English language, 2) contain peer based teaching (as defined previously) 3) focused on ethical or professionalism learning outcomes 4) included exclusively undergraduate healthcare students as study subjects (both peers and learners). Articles were selected by TH, SA and FK through a systematic search of electronic databases. Selected databases were MEDLINE, EMBASE, CINAHL, SCOPUS, and The Cochrane Library. Search terms were text word based and applied to all included databases. A wide range of closely related search terms were utilised and combined in our search design. The use of similar search terms (e.g. ‘peer’ vs ‘peer-’) was helpful in picking up relevant literature with subtle or nuanced differences in terminology. Search terms are listed in full in demonstrating our reproducible and systematic approach. Search terms were 1) peer, peer-, peers, peer peer, peer-peer, near-peer, near peer 2) student(s), medical student(s) 3) teach, teacher, teachers, teaching, teaches 4) educate(s), education 5) ethic, ethics, ethical, profession, professional, professionalism. These search terms were combined (1 Ω2 Ω3 Ω4 Ω5) in keyword searches of all databases in February 2014. Titles and abstracts were screened with relevant studies read to determine eligibility. Citations of selected articles were also screened in augmenting our search.

## Results

The search produced a total of 2532 papers (PubMed=821, Embase=435, CINAHL=187, Scopus=938, Cochrane Library=154). The titles of all articles generated were examined on the above inclusion and exclusion criteria, with abstracts of possible importance considered for inclusion. 65 articles of interest were identified (PubMed=20, Embase=12, CINAHL=3, Scopus=30, Cochrane Library=0). After removal of duplicates and further a screening of abstracts to ensure inclusion criteria were adhered to, an initial total of 23 articles were selected and read in full. Application of inclusion/exclusion criteria narrowed this to 10 in the final review. Finally, a citation search was conducted in which the selected articles were examined for cited material that could be potentially relevant for inclusion. No additional suitable articles were identified in this process. A summary of results is laid out in Table 1 – this includes all selected studies with summaries of intervention type, number of participants, study objectives and significant findings.

### Direct Peer-to-Peer Ethics Education

The earliest studies included in the reviews by Edelstein^16^ and Frisch^17^ centred on morality as a measurement of ethical education. In Edelstein’s study, 171 dietetic students undertaking three clinical programs of variable length and content were administered pre- and post- program questionnaires. Moral judgment was measured as part of the questionnaire using Rest’s Defining Issues Test (DIT), a measure of moral judgment. The authors found that programs fostering peer discussion of moral dilemmas facilitated moral growth more than those that did not emphasise this aspect. Furthermore, analysis of the results indicated significant increases (p<0.05) in moral development scores post-program, independent of course length.^16^ Frisch conducted a study amongst 52 nursing students to establish whether a value analysis teaching strategy of nursing ethics impacted students' moral development. Control and experimental groups were evaluated, with the experimental group given a framework for ethical reasoning. Like Edelstein, Frisch used Rest’s Defining Issues Test (DIT) in measuring moral development pre- and post-intervention in both groups. The results demonstrated a strong correlation between DIT score gains and self-report of peer discussion of ethical issues. Further Chi-square analysis showed that students who discussed ethical problems outside of class were more likely to advance on the DIT P-score (a measure of the level of principled thinking) than those who did not (p<0.05).^17^

Lin et al. conducted an eight-week study comparing conventional lecture-based teaching and (peer-peer) problem-based learning amongst 142 senior nursing students. An ethical discrimination scale was devised by an expert panel and administered pre-and post-intervention in measuring ethical education outcomes. Although both teaching methods produced improvement in ethical discrimination ability scores (p<0.05), students who received PBL teaching performed better than those in conventional lecture-based teaching (p<0.001). The authors suggest a peer-based format may offer learning advantages over traditional didactic teaching methods.^18^

Whilst Edelstein,^16^ Frisch^17^ and Lin et al.^18^ objectively measured the efficacy of peer-based teaching, other studies utilised a subjective approach, analysing student self-reporting of peer-peer ethics education.

Three studies involved round table discussion of relevant clinical ethics, collecting student-completed questionnaires in assessing the efficacy of their intervention. Fryer-Edwards et al. developed and evaluated an ethics based educational program called “Ward Ethics” in seeking to facilitate the professional development of clinical medical students through peer discussion of clinical ethical dilemmas. The program was considered successful, valuable or useful by 94% of students, with 83% feeling that the sessions helped them outline how to managed risks, mistakes and failures.^19^ Parker et al. carried out a similar study, utilising a modified teaching ward round model in finding cases of ethical interest to supplement round-table discussions. Sessions were evaluated using pre- and post- course questionnaires, with paired T-tests used to examine the results. Statistically significant improvements were observed in students' willingness to contribute to ethical discussions and raise concerns with seniors. Anecdotal reports revealed that participants appreciated having structured time to listen and learn from their peers.^20^

Cohn & Lie carried out a collaborative peer exercise in which first-year medical students in small groups were to design their own codes of ethics and identify primary values within their constructed codes. The aim of this single-session exercise was to help students identify important ethical values, to differentiate between personal and professional values, and to report the connections between values expressed in medical school oath and those expressed in the ethics and professionalism curriculum. Student evaluations and narrative feedback indicate that students enjoyed the opportunity to explore professional values, and that they found the peer-to-peer collaborative exercise to be a helpful educational guide.^21^

### Indirect Peer-to-Peer Ethics Education

Elliott et al. developed a more expansive intervention incorporating a 2-year course entitled ‘Professionalism and the Practice of Medicine’ into the first two years of a medical school curriculum. Ethical judgment formed a core component of the curriculum in which student led sessions, formal peer assessment and peer feedback were integrated. When evaluated, 81.2% of the participants directly attributed their better ethical judgment skills to the curriculum and its successful use of peer education, assessment and feedback.^13^

Camp et al. evaluated the effectiveness of delivering feedback in a group setting compared to an individual setting amongst a class of first-year medical students. Students completed weekly evaluations of themselves and their classmates regarding seven aspects of professionalism on a six-point Likert scale, and results of these were shared with the students either individually or as a group. Feedback was given both at the mid-point of the study (pre-intervention) and at the end of the study (post-intervention). Analysis and comparison of pre- and post-intervention professionalism scores indicated that both group and individual feedback were successful in improving total professionalism scores to a statistically significant extent. This indicates that peer-based feedback is useful in improving student professionalism.^22^

**Table 1:** Summary of results

Author (Year)	Intervention type and length	Number of participants	Study objectives	Significant findings
Camp CL,*et al., *(2010)	Weekly peer evaluations of professionalism with mid-point (3 weeks) and end point (6 weeks) feedback.	49 first year medical students	Comparing efficacy of peer-peer feedback delivered in a group feedback session vs. individual student instructor feedback session in a gross anatomy course.	Pre- (weeks 1-3) and post-intervention (weeks 4-6) Total Professionalism Scores (TPS) were derived. A paired T-test demonstrated that peer-peer feedback improved overall TPS (p= 0.032)
Cohn F,Lie D, (2002)	Medical student construction of their own codes of ethics with identification of primary values within their code design.	“Small groups” of first year medical students	1) To report connections between values expressed in the medical school oath and those expressed in the ethics and professionalism curriculum of first year medical students. 2) For students to identify important values and differentiate between personal and professional values.	Student evaluation, narrative feedback and faculty observation indicate that the intervention was educationally helpful and enjoyed by participants.
Edelstein SF, (1992)	Investigation of link between increased moral judgment development, and longer exposure to patient care/more peer discussion opportunities on ethical issues/more availability to trained ethics instructors	171 dietetics students	To compare moral judgement (asset out in Kohlberg’s theory of Moral Development) of students before and after training from internships (n=96),coordinated undergraduate programs (n=61) and pre-professional practice programs (n=14)	Rest’s Defining Issues Test (DIT) was used to measure the level of cognitive moral development pre- and post- intervention. A separate questionnaire was used to determine mode of ethics teaching, the ethics training of instructors and the importance of ethics training in their programs curriculum. Programs that foster peer discussion of moral dilemmas were found to facilitate moral growth more than programs that do not emphasise this aspect.
Elliott D,*et al.*, (2009)	A medical school curriculum branch entitled Professionalism and the Practice of Medicine (PPM) involving 40 two-hour sessions over the first 2 years of medical school. The curriculum incorporates a specific emphasis on ethical judgment and includes student led sessions, formal peer assessment and peer feedback.	First and second year medical student cohorts over 7 years	To develop and evaluate a longitudinal course in professionalism spanning the first two years of medical school.	Five administrations of university educational objective feedback forms revealed that 81.2% of students attributed gains in skill related to ethical judgment to the PPM curriculum. There was additional positive anecdotal feedback.
Frish ND, (1987)	Control and experimental populations (n=24 and 28) were either not given or given ethical decision making instruction and then evaluated via questionnaire at the beginning and end of the semester.	52 nursing students	To establish whether a value analysis teaching strategy of nursing ethics impacted students' cognitive moral development (as set out in Kohlberg’s theory of moral development).	Rest’s Defining Issues Test (DIT) was used to measure the level of cognitive moral development. A strong correlation was found between DIT score gains and self-report of peer discussion of ethical issues. Chi square test analysis showed that students who discussed their ethical problem outside of class were more likely to advance on the DIT P-score (a measure of the level of principled thinking) than those who did not (p<0.05).
Fryer-Edwards K, *et al*., (2006)	“Ward Ethics” – A program of peer discussions guided by clinical faculty mentors trained in ethics facilitation. A total of 24 ninety-minute sessions were held across 4 hospitals, during the first week of six-week medical and surgical rotations.	89 third-year medical students estimated across all the sessions	To develop and evaluate a ward based ethics educational program designed to facilitate the professional development of clinical medical students.	102 student-written evaluations were obtained after 24 sessions. 94% student responses rated the sessions as valuable, useful or successful. 98% saw the sessions as the most successful way to facilitate discussions of challenging experiences amongst peers. 83% felt the sessions helped them to outline how to manage risks, mistakes, or failures.
Lin C, *et al.,* (2010)	Senior nursing students were randomly assigned to lecture based teaching (control) (n=70) and problem based learning (experimental) (n=72) intervention groups over an 8-week period.	142 senior nursing students	To compare lecture-type conventional styles teaching medical ethics with problem based learning (PBL) methods.	Ethical Discrimination and Learning Satisfaction scales were devised and administered. Ethical discrimination was measured pre- and post- intervention. Both interventions produced and improvement in nursing ethical discrimination ability scores (p<0.05) Students who received PBL performed better than those who received conventional teaching (p<0.001).
Nofziger AC, *et al.*, (2010)	Online peer assessments are completed during the second and third year of the course as part of the university curriculum. Students then completed an online questionnaire regarding their experiences of peer-assessment. Narratives were coded into themes by two members of the research team.	101 second-year medical students and 83 fourth-year medical students	To investigate which types of peer feedback are the most memorable for students and the transformations students experience as a result of peer assessment.	The authors analysed responses using mixed and qualitative-quantitative methods. There were 138 responses in total. 68 from fourth-year class, and 70 from second-year class. 65% reported important transformations in awareness, attitude of behaviour as a result of peer assessment.
Parker L,*et al.,* (2012)	A modified teaching ward round model with students supplying de-identified cases of ethical interest for round-table discussion in 4 one-hour sessions. Sessions were evaluated using a questionnaire after the first and last ward round.	60 fifth year medical students across four campuses	To assess the efficacy of clinical ethics ward rounds as an ethics education intervention.	Sessions were evaluated using a questionnaire after the first and last ward round. Data from 47 students who attended three or more ward rounds were used. Statistically significant improvements were observed in students' willingness to ‘contribute to ethics discussions’ and their ‘confidence to raise concerns with supervisors’ (P<0.05). Anecdotal comments suggest students enjoyed the methodology and relevance of the educational intervention.
Varga- Atkins T,*et al.,* (2010)	An online wiki was made available to 4 problem-based (PBL) groups. Students used this to share resources or ask questions relating to the objectives on professional development. Qualitative feedback was collected through a small-scale student survey, and four focus group sessions.	32 first-year undergraduate medical students	To establish whether use of wikis (collaborative websites) and whether they could enhance medical students' development of professionalism.	Only 25% (8 students) gave feedback via the questionnaire regarding the use of wikis for developing professionalism. However, 75% (24 students) participated in focus group feedback. Students identified the benefits of wikis: ease of sharing information/resources, enhancement of face-face PBL meetings and improved confidence with regard to achieving learning objectives.

Nofziger et al. conducted a study in which 101 second- and 83 fourth-year medical students completed an online reflection regarding their experiences of peer assessment. The aim of the study was to investigate what types of peer feedback are the most memorable for students, and what students learn from such assessments. 67% of students found peer assessment helpful, and 65% reported important transformations in awareness, attitude or behaviour, as a result of the intervention.^23^

Varga-Atkins et al. utilised an online forum away from the classroom in their study. They sought to establish whether online wikis could enhance the professional development of 32 first-year medical students through online peer-to-peer contact. Qualitative feedback was acquired through surveys and group sessions. Students identified the wikis as useful, as it facilitated their ease of sharing information or resources, and improved confidence in achieving their learning objectives.^24^

## Discussion

The limitations of our method must be acknowledged before discussion of our conclusions. In focussing our search strategy we have limited the scope to reviewed published literature only, omitting potentially relevant forms of grey literature such as conference proceedings and expert opinion. Additionally, given, the heterogeneous nature of the studies the team decided not to perform any formal quality analysis. We recognise that this makes it challenging to determine the values of the individual study outcomes. In spite of these limitations, the cohort of studies identified, when taken collectively, offer some tentative conclusions and point towards a topic requiring further exploration. Furthermore, given the narrative nature of this review, the methodology employed to elicit relevant literature is both systematic and easily replicable.

The most surprising discovery from our search process was the relative lack of studies identified in which peer-based ethics education is investigated. Ethics teaching is a core component of many healthcare training curriculums and a topic every professional should be knowledgeable about, given the high standards of behaviours and attitudes expected in practice.^3^ Given that ethics education lends itself to discussion and sharing of ideas rather than prescriptive learning of model answers, small group based sessions amongst peers seems intuitively ideal as a teaching format. For this reason, the low total number of studies that met the inclusion criteria is by itself an interesting outcome. Furthermore, many of the studies included were not measuring peer-based interventions per se; for example Lin et al. set out to compare lecture-based teaching with PBL methods (which included peer discussion).^18^ Therefore, the benefits of the PBL may well have been as a result of the differing pedagogy, rather than by the use of peers. Additionally, the studies are highly heterogeneous in pedagogy, stage and profession of healthcare students, and aims of their intervention. Given the ubiquity of these limitations, a strong case can be made for higher quality research on this topic. Indeed, only in three papers^16^^-^^18^was objective measurement of learning outcomes measured, the remainder of studies relying on student reporting in drawing conclusions. These limitations certainly diminish the gravitas with which conclusions are made.

However, a number the studies reviewed suggest that peer-based teaching can be employed successfully in a number of settings. The lack of peer-based ethics education studies suitable for review may not accurately reflect the extent of its use in healthcare curricula, which we as authors feel is likely far more widespread than our literature search suggests. If this is true, our review certainly demonstrates a lack of understanding of the impact of this teaching methodology. Reasons for the limited use of peers in teaching ethics may reflect the importance of ethics as a subject and a paternalistic approach taken by institutions, given that peer-educator ethical values may differ from the ideals of the university. Equally, the reasons behind this may be relatively benign, with “safe” didactic teaching utilised in minimising the staff, time and space required to deliver ethics teaching. Nonetheless, the positive outcomes of the small number of studies reviewed and the potential benefits of expanding peer-based ethics teaching certainly warrant further exploration of the methodology by the medical community. Future studies on the matter should not only seek to ascertain student satisfaction outcomes, but also objective measurement of intervention efficacy as an educative methodology. Our literature search was useful in highlighting the potential benefits and drawbacks of the utilisation of a peer-based ethics teaching approach.

An important benefit alluded to throughout many of the included studies was the apparent high levels of student satisfaction produced by the peer-based approach. In an increasingly consumer-driven education market, an attractive course program is vital in enhancing student satisfaction statistics and in attracting the highest calibre students. One might imagine that solidifying the evidence relating to peer-based teaching efficacy would further strengthen the appeal of such teaching and propel the methodology into prominence.

Indeed, part of future study design on the topic may include studies similar to those conducted by Varga-Atkins et al.^24^ In an increasingly technology driven education market, students may value the benefits offered by an online format of peer-based ethics teaching. The convenience of distance-based learning alone is a large pull factor and its efficacy compared to traditional classroom based approaches is comparable to that of traditional education in terms of grades and attitudes.^25^^-^^26^ However, questions still remain as to whether this format is viable in ethics teaching. There are certainly barriers that need to be overcome relating to privacy and the potential for confidential information to be open to access from unauthorised parties. Such issues emerge in the implementation of any online educational scheme and although not insurmountable, it may explain why healthcare curriculums have not made widespread use of internet-based peer-education schemes.

An obvious drawback to peer-education is the necessity to source a pool of willing peer-educators, who may be under time pressures or see little value in teaching their juniors, and the challenges of re-training peers in order to maintain this pool. However, with teaching becoming an ever-important skill for the healthcare professional,^27^ and with studies linking peer-educators to higher achievement,^11^ the rewards on offer for motivated peer-educators are enticing. An alternative approach would involve a programme that mandates peer-peer teaching as part of the programme for older students, although evidently this might well reduce the quality of educators.

A number of less explicit benefits and drawbacks to peer-based ethics teaching also exist, although these were not drawn out in the studies reviewed. The educational benefits extend beyond the curriculum with regard to factors such as positive role modelling. Furthermore, there is likely to be a perceived safety in discussing complex ethical dilemmas with small group of peers rather than in lecture based settings with more intimidating academics/lecturers. However, the peer-based format can easily lead to a variable consistency in teaching and this lack of control of taught materials could deter institutions from utilising the methodology, instead opting for a more paternalistic approach. We would argue that the format lends itself to educators being challenged by their students; a factor likely to nullify any potential negative outcomes.

## Conclusions

Our study sought to establish the extent to which the medical community values peer-peer teaching of ethics as a teaching methodology. Whilst the limitations in both volume and quality of reviewed literature must be acknowledged, there was consensus throughout the literature that suggested peer-to-peer ethics teachings is both valuable and enjoyable for participants. This suggests there may be potential in further utilising this pedagology in teaching ethics.

Our findings demonstrate that further objective study is required in solidifying the widespread advocation of such an approach within the medical community. In particular, the medical community would benefit from objective data demonstrating the efficacy of peer-based ethics education in comparison to other teaching methodologies. Currently, such studies are lacking from the literature, and investment in such research is likely to be fruitful in paving the way for evidence-based educational reform. Furthermore, future studies would benefit from a large sample size and concurrent acquisition of multiple sources of feedback within their methodology (including views from peer-teachers, peer-learners and objective exam scores), in order to effectively evaluate the value of peer based ethics teaching. The inclusion of such studies within the literature is likely to provide a clear mandate for reform amongst the medical education community, with benefits reaped in terms of educational efficacy and student satisfaction.

### Acknowledgments

The authors wish to thank Megan C Schwarz for her invaluable assistance throughout this process and both Dr Gareth Owen and Amy M Ward for reviewing earlier versions of the manuscript.

### Conflict of Interest

The authors declare that they have no conflict of interest.
